# Inappropriateness of Medication Prescriptions to Elderly Patients in the Primary Care Setting: A Systematic Review

**DOI:** 10.1371/journal.pone.0043617

**Published:** 2012-08-22

**Authors:** Dedan Opondo, Saied Eslami, Stefan Visscher, Sophia E. de Rooij, Robert Verheij, Joke C. Korevaar, Ameen Abu-Hanna

**Affiliations:** 1 Department of Medical Informatics, Academic Medical Center, University of Amsterdam, Amsterdam, The Netherlands; 2 Netherlands Institute for Health Services Research (NIVEL), Utrecht, The Netherlands; 3 Department of Geriatrics, Academic Medical Center, University of Amsterdam, The Netherlands; The University of Edinburgh, United Kingdom

## Abstract

**Background:**

Inappropriate medication prescription is a common cause of preventable adverse drug events among elderly persons in the primary care setting.

**Objective:**

The aim of this systematic review is to quantify the extent of inappropriate prescription to elderly persons in the primary care setting.

**Methods:**

We systematically searched Ovid-Medline and Ovid-EMBASE from 1950 and 1980 respectively to March 2012. Two independent reviewers screened and selected primary studies published in English that measured (in)appropriate medication prescription among elderly persons (>65 years) in the primary care setting. We extracted data sources, instruments for assessing medication prescription appropriateness, and the rate of inappropriate medication prescriptions. We grouped the reported individual medications according to the Anatomical Therapeutic and Chemical (ATC) classification and compared the median rate of inappropriate medication prescription and its range within each therapeutic class.

**Results:**

We included 19 studies, 14 of which used the Beers criteria as the instrument for assessing appropriateness of prescriptions. The median rate of inappropriate medication prescriptions (IMP) was 20.5% [IQR 18.1 to 25.6%.]. Medications with largest median rate of inappropriate medication prescriptions were propoxyphene 4.52(0.10–23.30)%, doxazosin 3.96 (0.32 15.70)%, diphenhydramine 3.30(0.02–4.40)% and amitriptiline 3.20 (0.05–20.5)% in a decreasing order of IMP rate. Available studies described unequal sets of medications and different measurement tools to estimate the overall prevalence of inappropriate prescription.

**Conclusions:**

Approximately one in five prescriptions to elderly persons in primary care is inappropropriate despite the attention that has been directed to quality of prescription. Diphenhydramine and amitriptiline are the most common inappropriately prescribed medications with high risk adverse events while propoxyphene and doxazoxin are the most commonly prescribed medications with low risk adverse events. These medications are good candidates for being targeted for improvement e.g. by computerized clinical decision support.

## Introduction

The elderly population is increasing, resulting in a concomitant increase in chronic diseases and functional impairment [1]. Moreover many of the elderly persons suffer from co-morbid conditions and disabilities that necessitate multiple medications or polypharmacy [2], [3].

Adverse drug events are common in ambulatory care settings [4] and up to 35% of high risk older outpatients develop preventable adverse drug events [5]. One cause of preventable adverse drug events is the prescription of inappropriate medications. Inappropriate medication prescription (IMP) has been defined as the prescription(s) that introduce(s) a significant risk of an adverse drug related event when there is evidence for an equally or more effective alternative medication [6]. It can also be described as the failure to achieve the optimal quality of medication use [7]. IMP has been classified as underprescribing, misprescribing or overprescribing [8]. Several factors increase the risk of IMP to elderly persons, including physiological changes like reduction in renal and hepatic function, both of which are detrimental of drug metabolism and disabilities like visual and cognitive decline.

Aparasu *et al*. [9] and Gallagher *et al*. [10] reviewed, in 2000 and 2006 respectively, the incidence of IMP in elderly persons. Both reviews included studies with elderly persons in any healthcare setting from community dwelling elders to nursing home residents. Since then the criteria of assessing IMP have been revised and new medication list based tools have been developed [11]–[14]. In addition, there is lack of a detailed review that compared the incidences of IMP within specific pharmacotherapeutic classes. Such a review is necessary to allow for the development of interventions that target the improvement of prescription of specific medications.

The aim of this systematic review was to identify and summarize published studies on IMP in elderly in primary care in order to quantify its extent in elderly persons, and to identify medications for which interventions may be implemented to improve medication prescription quality.

## Methods

### Data Sources and Searches

**Figure 1:**
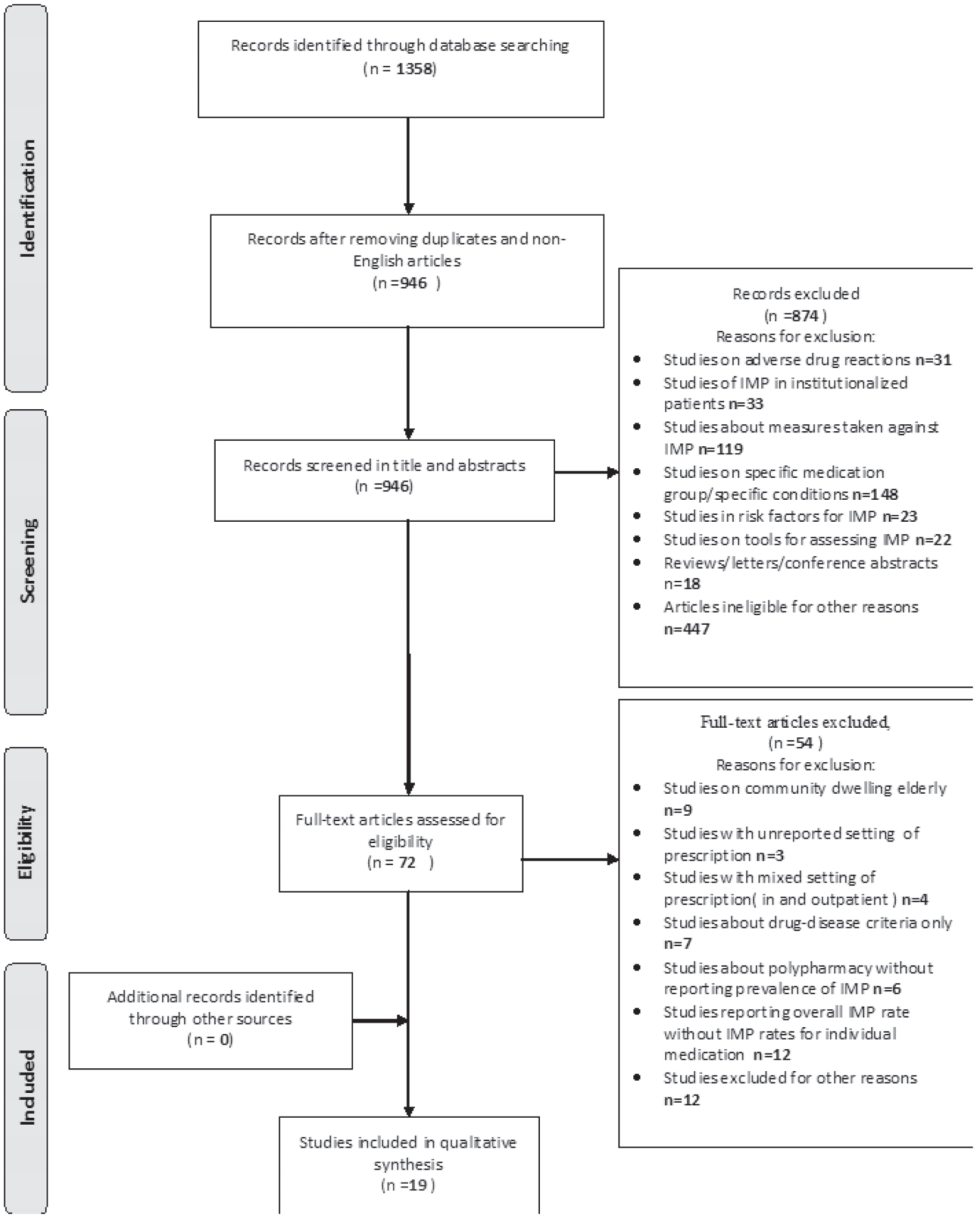
Article selection flow diagram.

We searched for relevant English articles using MeSH terms and keywords in title and abstract in the Ovid EMBASE (1980–8^th^ March 2012), Ovid-Medline and Ovid Medline In-Process (1950 to 8^th^ March 2012). The final literature search was performed on 8^th^ March 2012. Figure 1 shows the search strategy and its corresponding flow chart.

The search included terms related to elderly or geriatric persons, medications, prescription, appropriate prescription, primary care, ambulatory care, general practice, office practice or outpatient care as shown in Box S1. Duplicate articles found in both two databases were removed. We screened the references of the identified papers as supplementary search.

### Study Selection

Two reviewers independently screened the titles and abstracts of the retrieved articles. Inclusion was limited to studies that defined the elderly as persons of 65 years and older who received prescriptions in the primary care setting.

In this review, we only included papers that explicitly defined the setting of prescription as outpatient clinics, office practice, general practice and primary health care clinics. Studies that included institutionalized patients and community dwelling elderly without indicating the specific setting in which the prescriptions were prescribed were excluded. In addition, studies which did not report the clinical setting of the study were also excluded (see checklist S1).

Our analysis was limited to studies that reported IMP using drug-age criteria, which belong to the *unconditionally inappropriate medication prescription (IMP)* for persons 65 years old or older. Studies that reported IMP based on *drug-disease criteria* alone were not included due to heterogeneity of reporting in literature. For studies that evaluated IMP using the Beers criteria, we only included data indicating the rate of IMP measured independent of existing medical conditions and without any restrictions concerning dosage or duration of use, to allow for comparability of IMP with studies that did not use the Beers criteria. The principle of unconditionally inappropropriate prescription holds true for most of the list based criteria used for assessing IMP. Studies that reported only a single medication were excluded as well as studies that only described medication prescription for a specific disease group of elderly persons such as dementia patients.

To be included, studies must have also reported the rate of IMP for individual medications. This requirement allows for comparability of IMP rates of individual medication across different studies. Studies that reported rate of IMP after aggregating prescription by pharmacological classes, for example cardiovascular drugs, were excluded since it was impossible to find out which individual medications were included.

Discrepancies between the two reviewers were resolved by consensus involving a third reviewer.

### Data Extraction

The two reviewers extracted data from the selected articles on the following items: country; source of data used and instrument of assessing the appropriateness of medication prescription. Additional data extracted included the number of patients involved in each study and data on the rate of IMP. In the studies where more than one instrument was used to measure the rate of IMP, the rate estimated by the latest Beers criteria was used to compare studies. When repeated measurements were reported, we used the most recent rate of IMP. We calculated weighted rates of IMP for individual medications in studies that reported rates of IMP in males and females separately.

Discrepancies between these two reviewers were again resolved by consensus involving the third reviewer.

### Data Synthesis and Analysis

We calculated the median and ranges of the overall rates of IMP for all medications reported in a study. We compared the overall rates of IMP per country and source of data. For each study that assessed IMP with more than one instrument, we compared the incidences of IMP among them.

Subsequently, we grouped all individual medications reported in the studies into their Anatomical Therapeutic and Chemical (ATC) class. The ATC is a World Health Organisation (WHO) hierarchical standard for classifying medications based on the anatomical organ or system on which they act, the therapeutic group and chemical composition of the active ingredient [15]. We classified medications at the therapeutic (T) level of the ATC classification. We considered the following eight major therapeutic classes: analgesics and non-steroidal anti-inflammatory drugs (NSAIDs), sedative hypnotics, anticholinergics and antihistamines, antihypertensives, muscle relaxants, antidepressants, antiarrhythmics and anticlotting medications. Medications which do not belong to the above 8 therapeutic classes were not included for practical purposes.

For each medication in a therapeutic class, we calculated the median and range its IMP rate among all studies reporting on it. Additionally, each medication was labelled as high risk (H) or low risk (L) for adverse events to allow analysis at the level of a medication’s adverse event risk profile. This risk categorization was derived from the Beers criteria that distinguish between IMP with potentially low and high severity of adverse outcomes [6].

## Results

Out of 946 articles screened 19 met the inclusion criteria for detailed analysis. Table 1 lists the included articles. The studies were conducted in 11 different countries. Seven studies were performed in the United States of America and eight in Europe. Two studies were conducted in Italy while one study was conducted in each of the following EU countries: Germany, The Netherlands, Ireland, Norway, Portugal, and the United Kingdom. Two studies were performed in Taiwan while one study was conducted in Iran and India respectively.

**Table 1:** List of included articles.

No	Author	Year	Country	Data Source	No. Patients	Criteria	Overall IMP rate
1	Goltz [27]	2012	Germany	Insurance data	12513584	Beers 2003	2.90
2	Ghadimi [28]	2011	Iran	Insurance data	2041	Beers 2003	30.0
3	Zaveri [29]	2010	India	Prospective data	407	Beers 2003	23.6
4	Maio [30]	2010	Italy	Others	91741	Modified Beers 2003	25.8
5	Ryan [22]	2009	Ireland	Practice data	1329	Beers 2003	18.3
6	Lai [31]	2009	Taiwan	Insurance data	2133864	Beers 2003	19.1
7	Lin [32]	2008	Taiwan	Insurance data	5741	Beers 2003	23.7
8	Wilde [33]	2007	UK	National health data	162000	Beers 2003	32.2
9	Bierman [34]	2007	USA	National health data	965756	Zhan	15.6
10	Maio [35]	2006	USA	Prospective data	100	Beers 2003	25.0
11	De Oliveira [20]	2006	Portugal	Prospective data	213	Beers 2003	38.5
12	Maio [36]	2006	Italy	Insurance data	849425	Beers 2003	18.0
13	Pugh [37]	2006	USA	National health data	1096361	HEDIS HRME	19.6
14	Van der Hooft [21]	2005	Holland	National health data	25258	Beers 2003	20.0
15	Pugh [19]	2005	USA	National health data	1265434	Others	33.0
16	Curtis [38]	2004	USA	Insurance data	765423	Beers 1997	21.0
17	Aparasu [39]	1999	USA	National health data	NA	Beers 1997	4.45
18	Straand [17]	1999	Norway	Prospective data	16874	MRPS List	13.5
19	Aparasu [40]	1997	USA	National Health data	NA	Beers 1991	5.04

### Study Description

Eight different tools were used to assess the IMP rates. Beers based criteria were used in 15 of 19 studies: one study used Beers 1991, 2 studies used its 1997 version (Beers 1997), 11 used its 2003 version, (Beers 2003) and one study used modifications of Beers 2003. Other instruments used for assessing medication prescription included: Zhan’s criteria (1 studies) [16], More & Romsdal Prescription Study (MRPS) list (1 study) [17]. One study utilised the Health Plan Employer Data and Information Set (HEDIS) criteria [18]. One study did not explicitly mention the instrument used for assessing the quality of prescription [19].

Four studies used more than one instrument for assessing the appropriateness of medication use. Two studies [20], [21] that used the Beers 1997 and 2003 versions found consistently lower IMP percentages for the 1997 version: 27.7 vs. 38.5% and 18.5 vs. 20.0%. Pugh *et al*. found that Zhan’s criteria had had a low rate of IMP (0.8%) when compared to unidentified reporting criteria (33.3%) [19]. Ryan *et al*. compared Beers 2003 and the Screening Tool for Older Persons Prescriptions (STOPP) and found IMP rates of 18.3% and 21.4% respectively [22].

Six of the 18 studies (33.3%) used health insurance data, while 4 (22.2%) used prospectively collected data. Six studies (33.3%) used national health surveys and databases, and one study (5.6%) used general practice data. One study did not explicitly report the data sources used.

### Overall Inappropriate Medication Prescription Measures

The overall median rate of IMP among the elderly was 20.0% with an absolute range of 2.9 to 38.5% and interquartile range of 16.8 to 25.4%. In the seven studies from the United States of America, the median was 19.6% with a range from 4.5 to 33.3%. In the European Union the median rate of inappropriate prescription was 19.1% with a range of 2.9 to 38.5%.

Results for grouping prescriptions by the type of prescription quality assessment instrument were: for Beers 1997 the median IMP rate was 12.7% (range: 4.5 to 21.0%); for Beers 2003 the median was 23.6% (range: 2.9 to 38.5%); and for Zhan’s criteria, the only study reported a rate of IMP of 15.6%.

### Inappropriate Prescription within Therapeutic Classes

IMP rates markedly varied across and within individual therapeutic medication classes as shown in Table 2.

**Table 2:** Rate of inappropriate prescription of individual medications grouped by pharmacological classes divided according to the WHO ATC classification.

		Beers 1991	Beers 1997		Beers 2003											HD	MB	MR	OT	ZN		
MEDICATIONS	N	Aparasu [40]	Curtis [38]	Aparasu [39]	Goltz [27]	Ghadimi [28]	Zaveri [29]	Ryan [22]	Lai [31]	Lin [32]	Wilde [33]	Maio [35]	De Oliveira [20]	Maio [36]	Van der Hooft [21]	Pugh [18]	Maio [30]	Straand [17]	Pugh [19]	Bier-man [34]	Med	Range (LL- UL)
**Analgesic**																						
Indomethacin	**15**	7.0	6.5	3.86	0.40	6.2			6.5	0.2	0.55		0.9	3.9	0.5		1	0.2	1.4	2.04	1.40	0.20–7.00
Ketorolac	**6**								2.2		0			20.5	0.2	0.5	2.3				1.35	0.00–20.5
Meperidine	**6**				0.01				0.1		0.04					0.1			0.1	0.09	0.10	0.01–0.10
Naproxen	**5**							0.45		0.1	0.63	13	0.9								0.63	0.10–13.0
Pentazocine	**10**	0.96		0.44	0.01						0.01				0.1	0	0	0.04	0.1	0.02	0.03	0.00–0.96
Piroxicam	**2**										0.12		2.3								1.21	0.12–2.30
Propoxyphene	**9**	23.3		23.3			0.57				9.37				0.1	4.52		0.3	4.1	4.81	4.52	0.10–23.3
**Antarrhythmic**																						
Amiodarone	**11**				0.08	0.1	0.05	0.67	1.9	10.9	1.2	4	6.1	12.6	0.7						1.20	0.05–12.6
Digoxin	**4**									3	0.01		2.8				21.1		3.4		3.10	0.01–21.1
Disopyramide	**6**				0.01						0.05			0.4	0.1				0.1	0.05	0.08	0.01–0.40
**Anticholinergic**																						
Belladona alkaloids	**8**				0.03	0.05					0		0.5		0.3	0			0.1	0.02	0.04	0.00–0.50
Chlorpheniramine	**9**					0.7	1.09	0.07		0.7	1.21				0.1	2.11			1.8	2.26	1.09	0.07–2.26
Clidinium	**1**					3.1															3.10	3.01–3.10
Cyproheptadine	**7**				0.01	0.4					0.02				0.1	0.3			0.2	0.31	0.20	0.01–0.40
Dicyclomine	**7**		4.2			0.5	0.74					13				0.51			0.4	0.49	0.51	0.40–13.0
Diphenhydramine	**7**				0.03	4.4				0.5	0.02					3.52			3.3	3.8	3.30	0.02–4.40
Hydroxyzine	**11**		6.9		0.08	2.0		0.07			0.51	4	0.9		0.3	3.2			2.7	3.43	2.00	0.07–6.90
Hyoscamine	**3**															0.1			0.1	0.15	0.10	0.10–0.15
Oxybutinin	**9**		6.8		0.09	1.2		0.52		0.6	0.96	8.0			0.7				1.6	2.39	0.96	0.09–6.80
Propantheline	**3**															0			0.1	0.05	0.05	0.00–0.10
Promethazine	**6**				0.24	0.8									0.9	0.71			0.2	0.71	0.71	0.20–0.90
**Anticlotting**																						
Dipyridamole	**10**	18.6		12.56		0.7			0.1	36.1	0.24		1.4			0.6		0		0.58	0.65	0.00–36.1
Ticlopidine	**6**				0.03				2.6	1.2			8	18.3					0.4	0.51	0.86	0.03–18.3
**Antidepressant**																						
Amitriptiline	**16**	20.5	16.3	10.46	0.25	1.0	0.05	1.12	1.7	3.8	4.5		1.4	6.7	2		6.2	0.7	2.6	3.98	2.60	0.05–16.3
Doxepin	**7**		3.1						0.8	0.3	0.12				0.1				0.6	0.84	0.60	0.10–3.10
Fluoxetine	**7**					1.6			1.5	1.8	1.49	17	2.3		0.4						1.60	0.40–17.0
**Antihypertensive**																						
Clonidine	**8**				0.06		0.05	0.07			0.15	8		9.5	0.1		0.9				0.12	0.05–9.5
Doxazosin	**6**				0.32			6.47		15.7	3.91	4		23	0.5						3.96	0.32–15.7
Guanethidine	**2**								0.1		0										0.05	0.00–0.10
Methyldopa	**11**				0.07	1.4		0.07	0.2		0.2			0.5	0.1		0.6	1.23	0.1	0.1	0.20	0.07–1.40
Nifedipine	**8**				0.19	0.7		0.15	12.9	6	0.22		1.9			0.1	0.1				0.20	0.10–12.90
Reserpine	**3**										0								0.1	0.06	0.06	0.00–0.10
**Musclerelaxant**																						
Carisoprodol	**7**	0.50	3.6	1.49							0					0.2			0.1	0.16	0.20	0.00–3.60
Chlorzoxazone	**3**									6.1									0.1	0.2	0.20	0.10–6.10
Cyclobenzaprine	**5**	2.7	9.7	1.95												1.91			1.2	2	1.95	1.20–9.70
Metaxalone	**2**															0			0.1	0	0.05	0.00–0.10
Methocarbamol	**8**	1.0		0.42	0.01	4.0					0.7					2.21			1.6	2.41	1.30	0.01–4.00
Orphenadrine	**7**	1.8		0.99	0.01				3.1		0.05			1.6	0.1	0	0.7				0.70	0.00–3.10
**Sedatives**																						
Alprazolam	**2**										0								0.1		0.05	0.00–0.10
Chlorazepate	**4**				0.01						0.02		3.8		0.4						0.21	0.01–3.80
Chlordiazepoxide	**13**	6.0	2.4	8.7	0.07	3.0		0.3		1.6	0.24		0.9		0.2	0.4			0.4	0.44	0.44	0.07–8.70
Diazepam	**15**	11.6	10.6	30.05	0.43	1.6	0.05	2.7	21.3	2	2.74	13	11.7		2.8	1.5			1.2	1.57	2.74	0.05–30.0
Flurazepam	**10**	2.7		1.26	0.01	0.1			0.7		0.05				0.5	0.1			0.1	0.06	0.10	0.01–2.70
lorazepam	**4**					0.1		0.82			0.2								0.1		0.15	0.10–0.82
Meprobamate	**8**	3.5		0.93					0.1		0.1				0.1	0			0.1	0.01	0.10	0.00–3.50
Oxazepam	**2**										0								0.1		0.05	0.00–0.10
Temazepam	**3**							0.6			0.74	4							1		0.74	0.60–4.00
Triazolam	**3**							0.22			0								0.1		0.10	0.00–0.22

N: Number of studies.

HD: Health Plan Employer Data and Information Set (HEDIS) criteria.

MB: Modified beers 2003.

MR: More & Romsdal Prescription Study (MRPS) list.

OT: Other unspecified criteria.

ZN: Zhan’s Criteria.

ATC: World Health Organisation’s Anatomic Therapeutic and Chemical Classification.

Med: Median.

LL: Lower limit.

UL: Upper limit.

The four most commonly inappropriately prescribed medications were, in a decreasing order of IMP rate, were propoxyphene 4.52(0.10–23.30)%, doxazosin 3.96 (0.32–15.70)%, diphenhydramine 3.30(0.02–4.40)% and amitriptiline 3.20 (0.05–20.5)%.

### Non-steroidal Anti-inflammatory Drugs (NSAIDS)

Within the class of analgesic/NSAIDS, propoxyphene, which is a low risk analgesic medication, had the highest IMP median of 4.52% with range of 0.10 to 23.30%, while meperidine and pentozacine, which are high risk medications, had the lowest median of 0.1% and 0.03% (with range of 0.01 to 0.10% and of 0.00 to 0.44%, respectively).

### Antiarrhythmics

Dysopyramide had the lowest median rate of IMP 0.08(0.01–0.4)% among the antiarrhythmic medications while the digoxin was the most inappropropriately prescribed antiarrhythmic medication 3.10(0.01–21.1)%. Disopyramide is classified as a high risk medication while digoxin in a low risk medication.

### Anticholinergics

Diphenhydramine was the most inappropriately prescribed anticholinergic medication 3.30(0.02 4.40)% while belladonna alkaloids were the least inappropriate prescribed anticholinergic 0.04(0.0–0.50)%. Both diphenhydramine and belladonna alkaloids are high risk anticholinergic medications.

### Anticlotting Medications

IMP was reported for two anticlotting medication as follows dipyridamole 0.65(0.00–36.1)% and ticlopidine 0.86 (0.03–18.3)%. Dipyridamole is a low risk medication while toclopidine is a high risk anticlotting agent.

### Antidepressants

Doxepin was the least inappropriately 0.6(0.10–3.1) % prescribed antidepressant medication while amitryptiline 3.2(0.05–20.5)% was the most inappropriately prescribed. Both antidepressants are high risk medications.

### Antihypertensives

The median rate of IMP among the antihypertensive medication was lowest with guanethidine, 0.05 (0.00–0.1)%. Doxazosin 3.96 (0.32–15.7) % was reported to be the most inappropriately prescribed antihypertensive medication. Guanethidine is a high risk antihypertensive medication while doxazosin is a low risk medication.

### Muscle Relaxants

Among the muscle relaxants, metaxalone 0.05(0.00–0.1)% had the lowest IMP rates while cyclobenzaprine 1.95 (1.20–9.7)% had the highest. Both muscle relaxants are high risk medications.

### Sedative Hypnotics

Diazepam 2.74 (0.05 30.05)% had the highest rates of IMP while alprazolam 0.05 (0.00–0.10)% and oxazepam 0.05 (0.00–0.10)% had the lowest rate of IMP among the sedative hypnotic medications. All sedative hypnotics investigated are considered to be high-risk medications.

## Discussion

In spite of increasing attention to the quality of medication prescription among elderly persons presenting to the primary care setting, there are still high overall rates of inappropriate medication prescription in primary care. This review found that one in five (20.0%) prescriptions to elderly persons is inappropropriate with marked variation of rates of IMP within individual therapeutic classes.

The overall prevalence of inappropriate prescription showed wide variations between 2.9 and 38.5%. Several factors may contribute to this variation. Different countries use different sets of medications due to registration issues. There is hence no universal list of medications and criteria for assessing the overall medication use by older patients. Even within the United States inappropriate medication use markedly differed, suggesting that there some systematic differences between practices may exist. The differences in the quality of prescribing across geographical regions have recently been highlighted [23]. Cost and purchasing system of medication is another probable reason for the medication choices made in prescription [23]. Moreover, local drug procurement policies and the structure of financing of medication are among the probable factors that may contribute to the differences in prescription patterns.

Consistent with our review, the review of Aparasu *et al*. in 2000 estimated that between 14.2% and 25% of elderly patients were exposed to IMP [9]. Our review found an IMP rate of 20.0%. Of note, 16 out 19 studies included in our review were published after the year 2000 and the overall rate of IMP seems to not have decreased considerably over the last 11 years despite the attention that has been directed on the subject of IMP among elderly patients. While Aparasu *et al*. included studies that were based only on Beers criteria, our study included also non-Beers criteria based studies which reported IMP based on individual medications. Gallagher *et al.* found IMP rates of 12% among community dwelling elderly and 40% among patients in nursing homes [10]. Patients who stay in nursing homes are likely to be exposed to higher rates of IMP, as shown by Gallagher *et al* than those patients who receive prescription in the primary care setting. Unlike Gallagher *et al.* who reviewed studies that evaluated IMP in all clinical settings, our review was limited to studies that included patients who received their prescription in a primary care setting. The overall rate of IMP (20.0%) that we report in this review falls within the range (12–40%) reported by Gallagher *et al*. A more recent review of IMP among the elderly, which was based on studies that utilised administrative database, reported an IMP rate ranging from 11.5–62.5%. This finding is consistent with our overall IMP rate (20.0%) given that 12 out of 18 studies that we included were performed using retrospective databases [24].

Unlike the three reviews described above, our study compared the rate of IMP within therapeutic classes of medication. The patterns of inappropriate prescriptions vary considerably within therapeutic classes. Some medications with high risk for adverse events such as diazepam and nifedipine have high prevalence of inappropriate prescription compared to other medications in their respective therapeutic classes. Prescription of high-risk medication exposes the elderly to frequent and severe adverse drug events. Alternative low risk medications should be prescribed when available. There is therefore a need to move towards interventions that can improve the quality of medication prescriptions among the elderly in primary care such as employing clinical decision support systems (CDSS). These systems can provide alerts during prescription based on medication prescription guidelines such as the Beers criteria. Good alert design which focuses on the relevant information to the physician can improve the effectiveness of these systems [25].

This review included various list based criteria for the measurement of IMP which are comparable to each other given that they report individual medications. Modifications that were made in the various versions of the Beers criteria involved inclusion and exclusion of individual medications on the list of medications inappropriate for elderly patients. It was therefore possible for us to compare the rates of IMP for individual medications between studies even if they used different versions of the Beers criteria. However, the overall rate of IMP was not comparable between studies that used different versions of the Beers criteria or other instruments to measure the rates of IMP.

We regard classification of medications as unconditionally appropriate for elderly patients over 65 years as suggested by the Beers criteria to be an over simplification of real clinical practice. Some medications such digoxin or doxazocin may still be used safely in patients who are older than 65 depending on their clinical conditions. Improvements in the definition of inappropriate medication prescription have been made based on drug-disease combination in the elderly.

Twelve studies (12 of 19) relied on secondary analysis of existing database sources such as National Health Service databases, general practice and insurance databases, which were developed for other purposes. Although previous studies have shown that insurance databases do not necessarily have high quality clinical notes documentation, they do generally have high quality of medication documentation due to their use for reimbursement purposes [26]. An outstanding drawback of insurance health databases is, however, that only insured patients are enrolled. There is patient selection bias in countries or regions where insurance companies do not have universal coverage. Furthermore, we speculate that quality assurance and incentive programs by some insurers may lead to better quality of care.

### Strengths and Weaknesses

A strength of our study is the analysis of medication prescription appropriateness within therapeutic classes based on an international standard. This classification mitigates the difficulty in comparing inappropriate medication prescription measures originating from studies with different sets of medication that is influenced by the availability of medications and local prescription practices. This analysis is useful to policy makers and clinicians when making choices between medications from the same therapeutic class. We also compared IMP in studies which utilised multiple instruments to measure quality of prescription.

We used extensive search criteria to capture the different ways inappropriate prescription and elderly are referred to in the published literature. Nevertheless our study maybe limited by publication bias of studies on inappropriate medication prescription. In addition, some of the excluded articles may be relevant but due to uncertainty about or lack of reporting about the setting of the prescription, they have been excluded.

Furthermore, our study was limited by the heterogeneity of the included studies. The number of patients included in the studies varied widely, which makes it difficult to estimate the overall prevalence of IMP. Heterogeneity in reporting of the rates of IMP limited our ability to completely compare rates of IMP for individual medication in all studies. Some studies reported fewer drugs than others. We believe that unavailability of some medications in some countries may have resulted in the differences in the sets of medications which were reported.

Although our study was limited to quantifying the extent of IMP among elderly patients, it would be important to understand the factors that predispose these patients to IMP. In addition, understanding the relationship between IMP and the incidence of adverse events to the patients will provide better guidance to the prescribing physician. Demonstration of the relationship between IMP and adverse events require the validation of the tools used to define IMP in the first place.

Future studies that investigate therapeutic intents and choices of medication among physicians may help further understanding of their prescribing behaviour. This may particularly aid in understanding how choices can be presented to physicians in primary care. Such studies can result in improvement strategies by computerized decision support.

### Conclusion

Despite intensified efforts to scrutinize and improve the quality of medication prescription among elderly persons in the primary care setting, inappropriate medication prescriptions are still common. Approximately one in five prescriptions to elderly persons is inappropropriate. Diphenhydramine and amitriptiline are the most common inappropriately prescribed medications with high risk adverse events. These medications are good candidates for being targeted for improvement e.g. by computerized clinical decision support. Focused and systematic interventions are needed to improve the quality of medication prescription in this patient group.

## Supporting Information

Checklist S1 **PRISMA checklist.**
(DOC)

Box S1 **Complete search strategy.**
(DOC)
