# Supplementary material for: Inappropriateness of Medication Prescriptions to Elderly Patients in the Primary Care Setting: A Systematic Review
Source: PLoS One. 2012 Aug 22;7(8):e43617. doi: 10.1371/journal.pone.0043617 (PMC3425478; doi:10.1371/journal.pone.0043617)
Supplement: Box S1 — Complete search strategy. (DOC) [file pone.0043617.s002.doc]

Box S1: Full search strategy

1. (elder* or senior* or geriatr* or (old* and adult*)).af.

2. (medication and error*).af.

3. (prescr* and error*).af.

4. (medication and safety).af.

5. (prescr* and safety).af.

6. (inappropriate* and prescr*).af.

7. (inappropriate* and medication).af.

8. 2 or 3 or 4 or 5 or 6 or 7

9. ((primary and care) or (office and practice) or (ambulat* and care) or (general and practice) or (outpatient* and care)).mp. [mp=ti, ab, sh, hw, tn, ot, dm, mf, dv, kw, nm, ps, rs, ui]

10. 1 and 8 and 9

11. remove duplicates from 10

12. limit 11 to english language
